# Round the Hospitals

**Published:** 1921-05-14

**Authors:** 


### ROUND THE HOSPITALS.

The Poor-Law Matrons' Association has nominated Miss Barton (Chelsea Infirmary), Miss Clark (Whipps Cross Hospital), and Miss Alsop (Kensington Infirmary) as candidates for the College Council elections. We understand the voting-papers will be posted to College members within the next few days, and it is important that the voters should carefully study the names before signifying their choice, so that their selection may really represent their considered desire.

A very interesting classification of the nursing staff is given in the report of St. Bartholomew's Hospital, just issued. From this it appears that the day ward staff totals 164, including twenty-eight sisters, an average of 3.4 beds to each nurse; the night ward staff is seventy-seven, including two superintendents, an average of 7.2 to each nurse. The rationing allowance of 6s. a week instituted during the war for ward sisters and others provided with partial board only has been raised to 20s. a week. The number of nurses certificated last year after three years' training was thirty-seven, making a total of 1,487 since the training-school was started in 1880. The governors of the hospital express their gratitude to the nursing staff for the high standard of their work maintained under great difficulties due to the shortage of nurses which prevailed during the year. That difficulty has now lessened, and the matron received over a thousand applications, which were most plentiful at the end of the year.

The scheme of amalgamation between the Lambeth Infirmary and the workhouse opposite, which the Lambeth Guardians have passed for submission to the Ministry of Health, includes provision of satisfactory accommodation for the nursing staff, and obviates the need for a new nurses' hostel at an estimated cost of £50,000. It is believed it could be brought into operation by the beginning of next year, whereas the hostel could not possibly be erected without long delay. In the course of the somewhat heated debate on the scheme, one of the Guardians declared that the sleeping quarters for the nurses at present were "horrible," and another that they "bordered on something shocking—they were not cubicles but hutches." Yet when the boarding-out system was tried all the probationers petitioned (in spite of the drawbacks in the sleeping quarters) to be allowed to come into the Home instead of sleeping out.

The Plymouth Guardians, it appears, have given notice of their intention to reduce the bonus of their indoor staff, including the nurses, by 50 per cent. A former sister of the Greenbank Infirmary writes an able letter to the *Western Morning News* to protest against lowering the nurses' salaries. She relates that the pay she received during the first four years at this institution was £8, £12 10s., £15, £20. "Would they consider, after that, a sister of several years' experience, with a salary of £45, with £28 10s. war bonus, paid too much?" We cordially agree with her conclusion, "Pay the nurses a living wages and cut the war bonus altogether." There will have to be a big battle on the part of the nurses everywhere if the bonuses which now bring their salaries to an irreducible minimum are threatened.

The War Office has issued an official statement of the deaths in the various Services of British Nurses between August 4, 1914, and January 1, 1920. They number 300—viz., Q.A.I.M.N.S. 6, the Reserve 105, T.F.N.S. 48, a total of 159 fully-trained nurses; and 141 V.A.D. nursing members. Of the fully-trained nurses 29 were killed or drowned through enemy action, 6 were killed or drowned accidentally, 51 died abroad, and 73 died at home. Of the V.A.D.s 16 were killed or drowned through enemy action, 5 were killed or

**ROUND THE HOSPITALS—**(*continued*).

drowned accidentally, 32 died abroad, and 88 died at home. It is a tragic record, but one which must deepen respect for the women who carried through their whole work, regardless of their lives, at the call of their country.

H.R.H. Princess Christian will open the new Club of the Royal British Nurses' Association, 194 Queen's Gate, S.W., on May 18, at 4 p.m.

Under the title of the Compassionate Sub-Committee, the Joint Nursing and V.A.D. Services Committee of the United Services Fund has set up a Committee to administer the grant that has been made by the latter fund for the benefit of ex-Service nurses—*i.e.*, naval, military, Territorial Joint War Committee, and V.A.D. members—who have suffered disability as a result of their war service. The Chairman of the Sub-Committee is Sir Napier Burnett, K.B.E., M.D., Director of the Hospital Services Department of the British Red Cross Society. The address of the Compassionate Sub-Committee is 19 Berkeley Street, W. 1, and all applications for assistance should be made in writing to the Secretary.

The Ladies' Linen League of the Royal Southern Hospital, Liverpool, celebrated its tenth anniversary recently by a Bazaar and Sale of Work in the new out-patient department of the hospital. In addition to a large variety of stalls, numerous side-shows, games and competitions, enthusiastically run by members of nursing staff, contributed largely to the enjoyment and success of the afternoon, and the performance of the police band which was kindly lent by the Chief Constable for the occasion greatly added to the gaiety of the occasion. Sir Robert Jones, K.B.E., C.B., F.R.C.S., who was welcomed by Mr. Lyon H. Maxwell (honorary treasurer of the hospital) and Mrs. John Rankin (president of the League), opened the Fair. Mrs. Lyon H. Maxwell, who is the honorary treasurer of the League, has had the satisfaction of a record year. Nearly 12,000 articles of linen and clothing were contributed to the hospital, and the members of the League are looking forward to the time when they will be able entirely to relieve the hospital of the cost of one of its most expensive departments. We congratulate the League on their good work and wish them a speedy accomplishment of their splendid aim.

The district nurses at Neath, who have been labouring, at very inadequate pay, to keep pace with the requirements of the Education Committee as regards school visiting, have withdrawn their notice to discontinue this branch of their work, pending a thorough reorganisation. Sixteen additional district schools are coming under the jurisdiction of the Neath Education Committee, and it is in contemplation to apply for a State grant and appoint whole-time school nurses. At the same time it is universally admitted that the Neath district nurses practically created this branch of work, and made themselves indispensable. We trust these nurses will have the first refusal of any new posts which may be created under the new system.

